# Oropouche Virus: Isolation and Ultrastructural Characterization from a Human Case Sample from Rio de Janeiro, Brazil, Using an In Vitro System

**DOI:** 10.3390/v17030373

**Published:** 2025-03-05

**Authors:** Ana Luisa Teixeira de Almeida, Igor Pinto Silva da Costa, Maycon Douglas do Nascimento Garcia, Marcos Alexandre Nunes da Silva, Yasmim Gonçalves Lazzaro, Ana Maria Bispo de Filippis, Fernanda de Bruycker Nogueira, Debora Ferreira Barreto-Vieira

**Affiliations:** 1 Laboratory of Viral Morphology and Morphogenesis, Oswaldo Cruz Institute, Oswaldo Cruz Foundation—Fiocruz, Rio de Janeiro 21040-900, RJ, Brazil; maycongarcia@aluno.fiocruz.br (M.D.d.N.G.); marcossilva@aluno.fiocruz.br (M.A.N.d.S.); lazz.ysm05@gmail.com (Y.G.L.); 2 Laboratory of Arboviruses and Hemorrhagic Viruses, Oswaldo Cruz Institute, Oswaldo Cruz Foundation—Fiocruz, Rio de Janeiro 21040-900, RJ, Brazil; ana.bispo@ioc.fiocruz.br (A.M.B.d.F.); nandanog@ioc.fiocruz.br (F.d.B.N.)

**Keywords:** Oropouche virus, ultrastructural studies, transmission electron microscopy

## Abstract

The Oropouche virus (OROV) is a segmented negative-sense RNA arbovirus member of the *Peribunyaviridae* family, associated with recurring epidemics of Oropouche fever in Central and South America. Since its identification in 1955, OROV has been responsible for outbreaks in both rural and urban areas, with transmission involving sylvatic and urban cycles. This study focuses on the characterization of an OROV isolate from a human clinical sample collected in the state of Rio de Janeiro, a non-endemic region in Brazil, highlighting ultrastructural and morphological aspects of the viral replicative cycle in Vero cells. OROV was isolated in Vero cell monolayers which, following viral inoculation, exhibited marked cytopathic effects (CPEs), mainly represented by changes in cell morphology, including membrane protrusions and vacuolization, as well as cell death. Studies by transmission electron microscopy (TEM) revealed significant ultrastructural changes, such as apoptosis, intense remodeling of membrane-bound organelles and signs of rough endoplasmic reticulum and mitochondrial stress. Additionally, the formation of specialized cytoplasmic vacuoles and intra- and extracellular vesicles emphasized trafficking and intercellular communication as essential mechanisms in OROV infection. RT-qPCR studies confirmed the production of viral progeny in high titers, corroborating the efficiency of this experimental model. These findings contribute to a better understanding of the cytopathogenic mechanisms of OROV infection and the contribution of cellular alterations in OROV morphogenesis.

## 1. Introduction

The Oropouche virus (OROV) was first isolated in 1955 from human blood samples in Trinidad and Tobago [1]. The transmission of OROV includes a sylvatic cycle, involving sloths, rodents, and non-human primates, whereas in the urban cycle, humans become the main host with transmission through the bite of infective bridges, with *Culicoides paraensis* as its primary vector [2]. In Brazil, the first isolation of the virus was achieved from blood samples of the species *Bradypus tridactylus* (sloth) and from a pool of *Aedes serratus* mosquitoes, in 1960 [3]. The following year, the isolation of OROV from human samples occurred during an outbreak in Belém, Pará, Brazil, with 11,000 cases reported, leading to the recognition of OROV’s epidemic potential [3].

Although historically overshadowed by other diseases, such as dengue and zika, Oropouche fever is the second most prevalent arboviral disease in Brazil [4]. Over the decades, the disease has been primarily reported in northern Brazil, especially in the Amazon region [5]. On the other hand, transmission outside this area went largely undetected by public health surveillance systems due to differential diagnosis issues [6,7]. Recently, Oropouche fever has emerged as a significant public health concern in Central and South America, particularly in Brazil, with an increasing incidence of cases [8]. From 2022 to 2024, there has been an alarming increase in both the geographical spread, with sustained transmission across non-endemic regions, and severity of outcomes [9,10].

The clinical symptomatology resulting from OROV infection resembles many of the other arboviral diseases circulating in Brazil, such as dengue and chikungunya, which hampers clinical diagnosis [11]. Commonly, initial symptoms include fever, headache, chills, malaise, myalgia, and arthralgia; however, in some cases, patients may develop more severe conditions, such as vomiting, bleeding, neurological signs and, in rare instances, death [12]. However, starting in 2024, there have been reports of fatalities linked to OROV infection among immunocompetent patients, with two documented cases of rapid progression to hemorrhagic symptoms leading to death within a few days in previously healthy individuals [9]. Furthermore, there are concerning reports of adverse pregnancy outcomes, including miscarriages and stillbirths, associated with OROV infection [13].

In July 2024, the Pan American Health Organization (PAHO) alerted two possible cases of vertical transmission in Pernambuco, Brazil, both of which resulted in fetal death, and the viral RNA was detected in multiple organs of the stillborns [14]. Even though these complications have been increasingly recognized in recent reports, there was prior evidence indicating that OROV was associated with pregnancy losses during an outbreak in Manaus, Brazil, between 1980 and 1981. Researchers from the Evandro Chagas Institute reported nine cases of OROV infection in pregnant women, in 1982, two of which resulted in spontaneous abortion [15].

These findings already suggested the potential for the vertical transmission of OROV, a phenomenon that remains poorly understood and warrants further investigation. Naveca and colleagues analyzed 382 OROV genomes from human samples obtained from northern Brazil and revealed the occurrence of several mutations in the circulating virus that may be related to fetal and neurological alterations [16]. Results like this indicate the unprecedented dispersal and evolution of OROV, highlighting its genomic dynamics and potential epidemiological impact.

OROV is a negative-sense RNA virus with a tri-segmented genome, belonging to the family *Peribunyaviridae* and the genus *Orthobunyavirus*. Orthobunyaviruses are enveloped viruses with an approximate diameter of 90–100 nm [17]. Their genome consists of three segments: small (S), medium (M), and large (L) [2]. The S segment encodes the nucleoprotein and non-structural proteins, the M segment encodes glycoprotein precursors that are cleaved into the Gn and Gc glycoproteins, and the L segment encodes the RNA-dependent RNA polymerase (RdRp). For OROV entry into host cells, pH acidification is required to induce conformational changes in the structural proteins Gn and Gc [18]. OROV entry into HeLa cells necessitates the presence of clathrin-coated vesicles, further supporting the role of acidification in facilitating viral entry into cells [19].

Viral isolation is often one of the initial and essential steps for various types of studies involving arboviruses. OROV has been isolated from samples of humans, non-human primates, and mosquitoes across different geographic regions and time periods [20,21,22,23]. However, although previous studies have demonstrated the isolation of OROV in cell cultures, the main alterations in mammalian cells, the detailed characterization of its replicative cycle, and the ultrastructural changes in infected cells remain poorly explored.

The aim of this study was to isolate OROV from the serum of a patient in mammalian cells, determine its cytopathic effects (CPEs), and characterize the main ultrastructural changes in infected cells. To this end, Vero cells were inoculated with OROV-infected supernatant and analyzed using bright-field microscopy and transmission electron microscopy (TEM). In addition to isolating OROV in mammalian cells, this study established a plaque assay titration protocol and provided a detailed description of the ultrastructural alterations observed in infected cells. These advances are fundamental for understanding the replicative cycle of OROV and represent a crucial step towards the development of drugs, treatments, vaccines, and diagnostic tools for this neglected virus.

## 2. Materials and Methods

### 2.1. Virus 

A blind infection with 100 µL of the original clinical sample was performed in a 25 cm^2^ culture flask containing Vero cell monolayers (ATCC CCL-81) at approximately 80% confluence, in high-glucose Dulbecco’s Modified Eagle Medium (DMEM/Gibco) supplemented with 2% fetal bovine serum (FBS/Gibco), 1% Pen-Strep (Gibco, Waltham, MA, USA), and 25 mM HEPES (Gibco). After 48 h (h) and the detection of CPEs through bright-field microscopy, the supernatants were collected and stored at −70 °C. Following collection, a Plaque-Forming Unit (PFU)/mL technique plaque assay was conducted. The sample used in this study was kindly provided by the Arbovirus and Hemorrhagic Virus Laboratory of the Oswaldo Cruz Institute (IOC), Oswaldo Cruz Foundation (Fiocruz), Rio de Janeiro, Brazil and this was collected in the Municipality of Piraí, state of Rio de Janeiro on 2 April 2024. The sequencing demonstrated that the virus belongs to lineage BR2015-2024 of *Orthobunyavirus Oropoucheense* (Accession ID: EPI_ISL_19491757 [GISAID EpiArbo]). The access to the genetic patrimony of the virus isolated is registered in the National System for the Management of Genetic Heritage and Associated Traditional Knowledge (SisGen A0C0D2F). This research was approved by the Ethics Committee of Oswaldo Cruz Institute (protocol number CAAE: 90249218.6.1001.5248). All experimental procedures were conducted in a biosafety level 3 (BSL-3) laboratory.

### 2.2. Viral Mass Production and Titration

OROV was propagated in Vero cells for 48 h at 37 °C in DMEM with high glucose, supplemented with 2% FBS, HEPES (25 mM), and antibiotics (penicillin and streptomycin, 100 U/mL). The multiplicity of infection (MOI) used for propagation was 0.01. Following propagation, the supernatant was collected, clarified of cellular debris by centrifugation at 700× *g* for 20 min at 4 °C, and subsequently stored at −70 °C.

The viral titer was assessed using a PFU assay with Vero cells. Confluent monolayers of Vero cells, grown in 12-well plates, were infected with serial dilutions of OROV and incubated for 60 min at 37 °C in a 5% CO_2_ atmosphere, with gentle agitation every 15 min. After incubation, non-adsorbed viruses were removed by washing with Phosphate-Buffered Saline (PBS). A semi-solid medium (1.6% carboxymethyl cellulose in 2× high glucose DMEM with 2% FBS) was then added to the wells. The plates were incubated at 37 °C in a CO_2_ atmosphere for 48 h. Following this incubation, the semi-solid medium was removed, and the cells were fixed with a crystal violet solution (containing 20% formaldehyde, 30% ethanol, and 1% crystal violet) for plaque enumeration. All experimental procedures were conducted in a BSL-3 laboratory.

### 2.3. Infection Kinetics

In order to analyze the morphological profile via bright-field microscopy, infection kinetics were assessed at 24, 48, 72, and 96 h post-infection (p.i.), using a MOI of 0.01 on Vero cell monolayers (ATCC CCL-81). Bright-field microscopy was employed at time points between 24 and 96 h p.i. using the EVOS XL Core imaging system, with results compared to control conditions (uninfected monolayers maintained for the same duration as the infected). For ultrastructural analysis by TEM, monolayers at 48 h p.i. were harvested and processed accordingly. Supernatants from all conditions were collected and titrated using the PFU/mL assay. All experimental procedures were conducted in a BSL-3 laboratory.

### 2.4. Processing Samples for Analysis by TEM

Based on our initial evaluation of the CPEs through bright-field microscopy, the time of 48 h p.i. was selected for ultrastructural evaluation. Cell monolayers were trypsinized and the suspended cells were fixed in 2% glutaraldehyde in 0.1 M sodium cacodylate buffer and in 1% buffered osmium tetroxide, respectively. Following fixation and post-fixation, the samples were dehydrated through immersion in increasing concentrations of acetone (15%, 30%, and 50% for 15 min each; 70% in 1% uranyl acetate for 30 min; 90% for 5 min; and twice in 100% for 10 min). Subsequently, the cells were embedded in epoxy resin and polymerized at 60 °C for three days [24,25]. Ultrathin sections (50–70 nm) were then obtained from the resin blocks using an ultramicrotome equipped with diamond knives. The sections were collected on uncoated copper grids with a mesh size of 300 and examined using a Hitachi HT 7800 (Hitachi, Tokyo, Japan) transmission electron microscope.

### 2.5. Quantitative Analysis of Mitochondrial Morphology

Mitochondrial structure was quantitatively assessed using TEM micrographs of Vero cell cultures. Initially, 20 micrographs containing OROV-infected cells and 20 micrographs with uninfected cells (control cells) were measured on mitochondrial density. Subsequently, the cross-sectional area and the dimensions of the minor and major axes were determined for 500 mitochondria per group (OROV-infected and control).

All variables were measured with the aid of an image analysis software, ImageJ software version 1.53t (NIH ImageJ, National Institutes of Health, Bethesda, MD, USA). The mitochondrial density was calculated as the number of mitochondria per square micrometer detected in each micrography. Both cross-sectional area and circularity index of mitochondria were quantified and presented as the individual values of 500 mitochondria per group. The circularity index was calculated as the ratio of minor to major axes, with a ratio closer to one indicating a more rounded shape.

A non-parametric test was deemed most appropriate, considering that the data did not exhibit a normal distribution and displayed relatively high variance, due to the naturally occurring pleomorphism associated with mitochondria. Specifically, the Mann–Whitney test was employed, with a significance level set at *p* < 0.05. Although this test is not exclusively reliant on medians, it places significant emphasis on their differences between groups, given that the median values have been highlighted in the results section. The distribution of data and the statistical test to compare both groups were assessed using GraphPad Prism software version 8.0.1 (GraphPad Software Inc., La Jolla, San Diego, CA, USA), which was also used to generate the graphs.

### 2.6. Viral Extraction and RT-qPCR

Viral RNA was extracted from 140 µL of Vero cell supernatant (72 h p.i.) using the QiaAmp Viral RNA mini kit (Quiagen, Hilden, Germany), according to the manufacturer’s instructions. The extracted RNA was reverse transcribed into cDNA and the genomic target amplified in a one-step reaction using the GoTaq^®^ Probe 1-Step RT-qPCR System (Promega, Fitchburg, WI, USA) on QuantStudio 5 Real-Time PCR Systems (Thermo Fisher Scientific, Waltham, MA, USA), according to the protocol described by Naveca [26]. Normal human serum and nuclease-free water were used as negative controls during RNA extraction and RT-qPCR.

## 3. Results

### 3.1. Morphological Analysis of Infected Vero Cells by Bright-Field Microscopy

**Figure 1:**
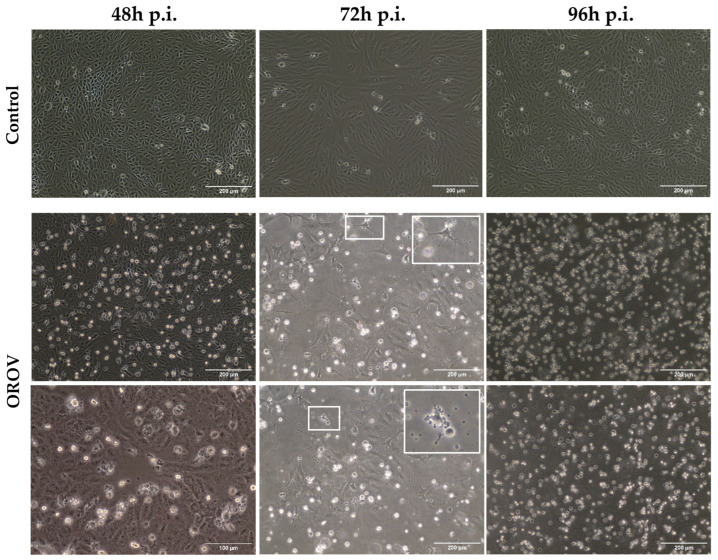
Monolayers of Vero cells in control conditions and infected with Oropouche virus (OROV) at 48, 72, and 96 h post-infection (h p.i.). Bright-field microscopy analysis revealed conformational changes in the membrane as the infection progresses, including the formation of projections and membrane partitioning (insets), which are indicative of apoptosis.

Infected cell monolayers already showed significant CPEs at the early time points analyzed. There was evidence of cellular debris, detached cells, and intercellular spaces as early as 48 h p.i. This phenomenon was absent in the control condition, supporting the assertion that the virus effectively infects the cells, proceeding the replication and inducing cell death (Figure 1). The supernatant of the Vero cell infected monolayer was collected 48 h p.i. Following a titration assay, the viral plaques were characterized, and the titer was established as 1.8 × 10^7^ PFU/mL.

From a morphological perspective, a more compartmentalized cell monolayer can be observed, suggesting a possible increase in the number of intracellular vesicles. At 48 h p.i., a subtle change in the morphological pattern of infected cells is noticeable, an effect that becomes more pronounced at 72 h p.i. The characteristic spindle-shaped pattern of the cells was lost, giving way to cells with membrane projections. In some cells, membrane compartmentalization was observed, indicating an apoptotic process. All the effects described at 48 h p.i. were exacerbated after 72 h. At later stages, a high level of cell death was evident, making it impossible to discern any morphological characteristics. This cell death could be observed in the fields corresponding to 96 h p.i., as indicated by the exclusive presence of cellular fragments that were freely floating in the cellular flask (Figure 1).

### 3.2. Ultrastructural Features of Cytopathic Effects Induced by OROV in Vero Cells

**Figure 2:**
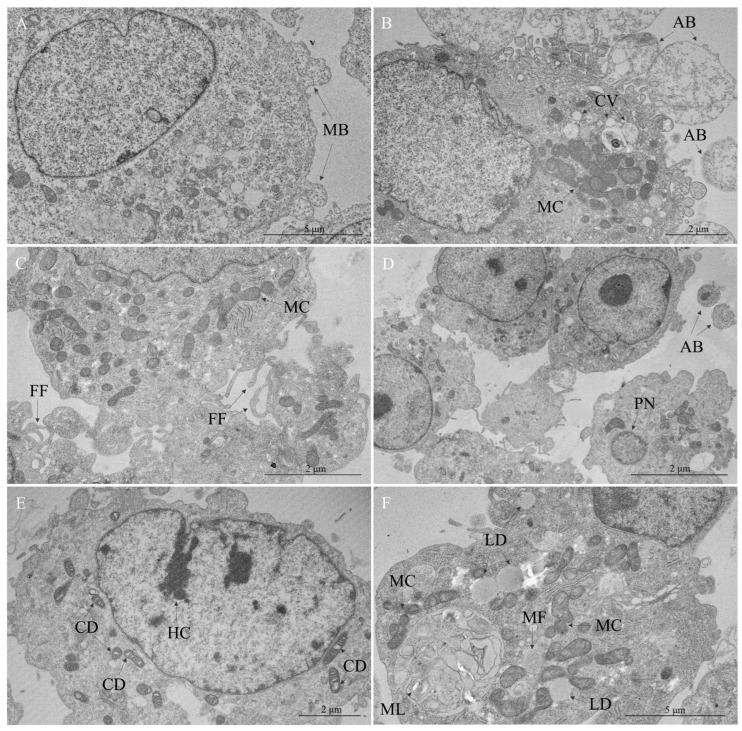
Cytopathic effects in Vero cells infected with Oropouche virus—cellular response and damage. (**A**) Uninfected Vero cell. Membrane blebbing (MB). (**B**–**F**) OROV-infected cells. (**B**) Apoptotic bodies (AB), cytoplasmic vacuoles (CV), mitochondrial clustering (MC). (**C**) Filopodia formation (FF), mitochondrial clustering (MC). (**D**) Apoptotic bodies (AB), pyknotic nucleus (PN). (**E**) Mitochondrial cristae disorganization (CD), hypercondensation of chromatin (HC). (**F**) Lipid droplets (LD), mitochondrial clustering (MC), myelin figure (MF), multilamellar bodies (ML).

No ultrastructural alterations were observed in uninfected Vero cells except for discrete plasma membrane blebbing in some cells (Figure 2A). In contrast, the infected cell culture exhibited the formation of large apoptotic bodies (Figure 2B) and filopodia (Figure 2C). The nuclear profile remained regular in most cells, except for a few shrunken pyknotic nuclei (Figure 2D) and chromatin condensation (Figure 2E). A significant clustering of mitochondria was observed, especially in regions rich in cytoplasmic vacuoles and multilamellar bodies, which were consistently found (Figure 2B,F). Mitochondrial swelling was another relevant change (Figure 2E,F), as well, as the presence of lipid droplets, constantly seen in mitochondria-rich microenvironments (Figure 2F).

**Figure 3:**
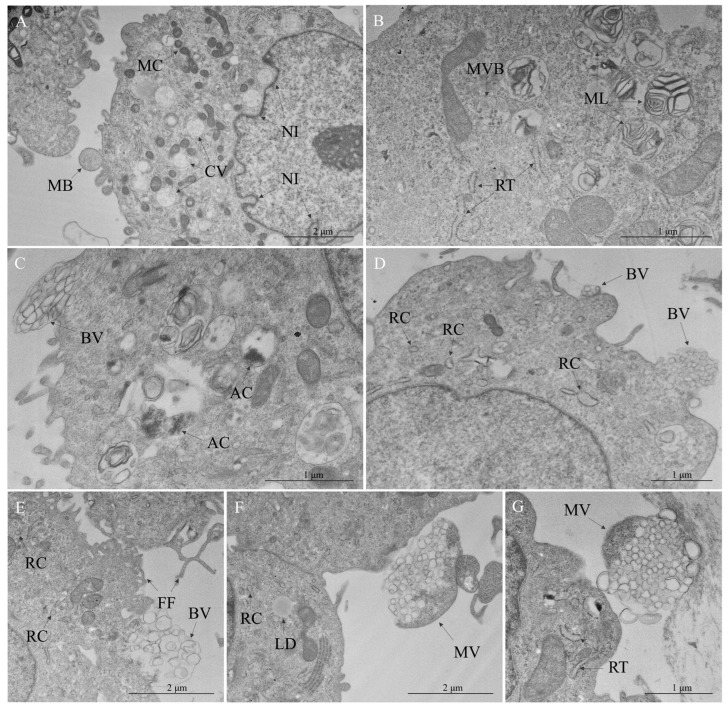
Cytopathic effects in Vero cells infected with Oropouche virus—membrane remodeling. (**A**) Cytoplasmic vacuoles (CV), plasma membrane blebbing (MB), mitochondrial clustering (MC), nuclear membrane invagination (NI). (**B**) Multilamellar body (ML), multivesicular body (MVB), rough endoplasmic reticulum (RER) thickening (RT). (**C**) Autophagy components (AC), budding vesicles (BV). (**D**) Budding vesicles (BV), RER cisterns (RC). (**E**) Budding vesicles (BV), filopodia formation (FF), RER cisterns (RC). (**F**) Lipid droplet (LD), multivesicular cargo (MV), RER cisterns (RC). (**G**) Multivesicular cargo (MV), RER thickening (RT).

Membrane rearrangement was a ubiquitous feature of OROV-infected cells. Many of the vacuoles were characterized by their proximity to other organelles, either in mitochondrial clusters or in the perinuclear region, resulting in nuclear membrane invagination (Figure 3A). In addition to these translucent vacuoles, the development of multilamellar bodies and multivesicular bodies (MVBs) (Figure 3B) was observed. In fact, the convergence of autophagy and endocytic pathways was detected, with the presence of components such as autolysosomes (Figure 3C). Different forms of vesicles were present, as in the budding of vesicles (Figure 3C,D) and multivesicular cargo (Figure 3F,G). Interestingly, the neighboring of these vesicles usually exhibited alterations of the rough endoplasmic reticulum (RER), such as thickening and the formation of cisterns (Figure 3D–G).

**Figure 4:**
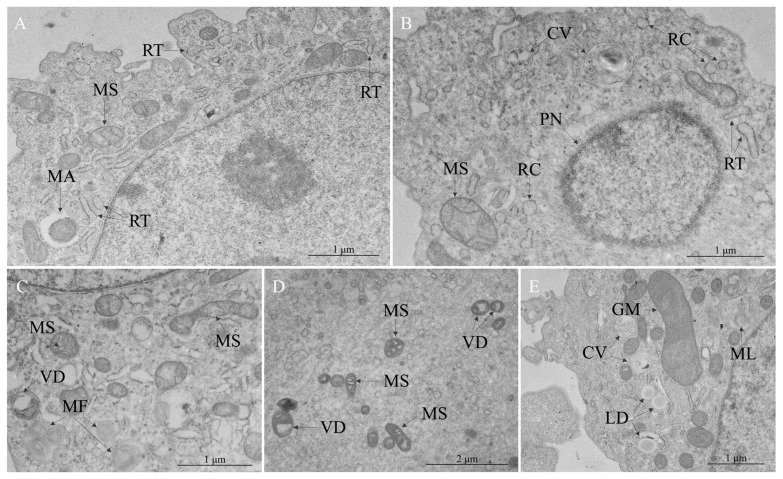
Cytopathic effects in Vero cells infected with Oropouche virus—rough endoplasmic reticulum (RER) and mitochondrial stress. (**A**) Mitophagy (MA), mitochondrial swelling (MS), RER thickening (RT). (**B**) Cytoplasmic vacuole (CV), mitochondrial swelling (MS), pyknotic nucleus (PN), RER cisterns (RC), RER thickening (RT). (**C**) Myelin figures (MF), mitochondrial swelling (MS), vacuolar degeneration of mitochondria (VD). (**D**) Mitochondrial swelling (MS), vacuolar degeneration of mitochondria (VD). (**E**) Cytoplasmic vacuole (CV), giant mitochondria (GM), lipid droplets (LD), multilamellar bodies (ML).

As shown, the RER exhibited several modifications compared to the control cells. The most common alteration in the RER was luminal thickening (Figure 4A,B). The formation of tubular structures represented an early stage of modification, in contrast to the establishment of cisternae (Figure 4B), a later phase, after which the ribosomes are lost. Another sign of RER stress was the formation of myelin-like figures (Figure 4C). Frequently, regions with the activation of the RER were observed near mitochondria. These organelles also showed alterations, such as swelling (Figure 4A–D), resulting in dilated cristae spaces and an electron-dense matrix and, in later phases, the vacuolar degeneration of mitochondria (Figure 4C,D) and mitophagy (Figure 4A). Most mitochondria presented a diminished size and more rounded aspect. Comparatively, some of them presented a significantly increased cross-sectional area, like giant mitochondria (Figure 4E).

**Figure 5:**
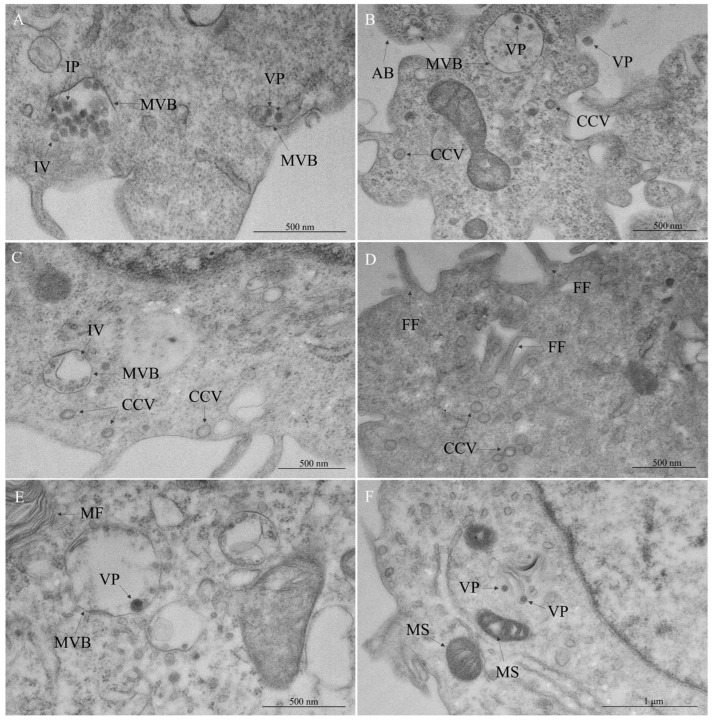
Cytopathic effects in Vero cells infected with Oropouche virus—viral replication insights. (**A**,**B**) Multivesicular body (MVB), viral particles (VP). (**A**) Immature viral particles (IP), intraluminal vesicle (IV). (**B**–**D**) Clathrin-coated vesicles (CCV). (**B**) Apoptotic body (AB). (**C**) Intraluminal vesicle (IV), multivesicular body (MVB). (**D**) Filopodia formation (FF). (**E**,**F**) Viral particles (VP). (**E**) Multivesicular body (MVB), myelin figures (MF). (**F**) Mitochondrial swelling (MS).

MVBs were consistently observed throughout our analysis (Figure 5A–C,E). Notably, particles resembling OROV at different stages of maturation were also found within these MVBs (Figure 5A,B), which simultaneously may contain intraluminal vesicles (Figure 5A,C). A smaller proportion of viral particles was detected outside cells, near to plasma membrane (Figure 5B), and associated with the Golgi apparatus (Figure 5F). Numerous clathrin-coated vesicles were identified in regions near to MVBs (Figure 5B–D) and regions of filopodia formation (Figure 5D).

**Figure 6:**
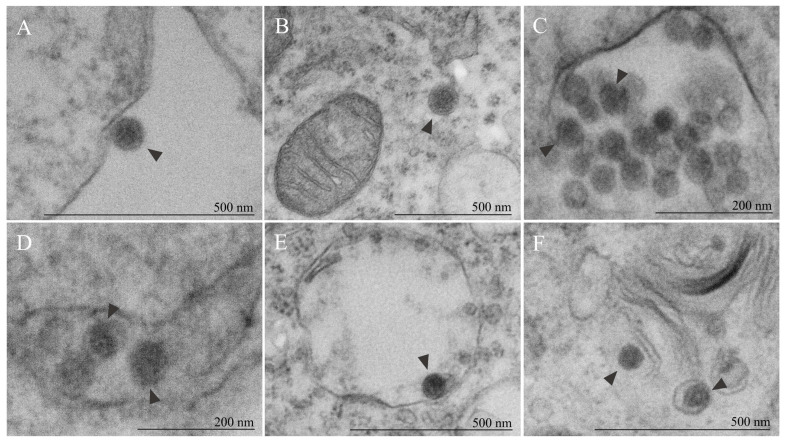
Morphology of Oropouche viral particles. (**A**) Viral particle (arrowhead) in extracellular space, adsorbed to the membrane surface. (**B**–**F**) Viral particles (arrowhead) into cytoplasm. (**C**–**E**) Morphological diversity noted within multivesicular bodies, showcasing particles at different stages of morphogenesis (arrowhead), leading to variations in size and electron density. (**C**) Reduced diameter observed in indicated particles (arrowhead) reflects incomplete particles. (**F**) Additional location of viral particles (arrowhead) within cytoplasm, associated with Golgi apparatus stacks. Inset: (**C**,**D**) Figure 5A. (**E**) Figure 5E. (**F**) Figure 5F. All particles exhibited a spherical shape, with variation in diameter indirectly reflecting maturation status of these particles.

The viral particles presented the typical characteristics associated with OROV and related viruses of the *Orthobunyavirus* genus (as spherical enveloped viruses, with a mean diameter of about 80 nm) during the analysis of Vero infected cell cultures. The particles were identified in different locations, including the extracellular environment (Figure 6A) and distinct compartments within the cytoplasm (Figure 6B–F). This distribution may account for the subtle morphological diversity observed, particularly within the MVBs, in which there was the simultaneous presence of particles at different stages of morphogenesis. Consequently, it resulted in variations in diameter and electron density (Figure 6C,D).

### 3.3. Morphometrical Assessment of Mitochondrial Structure

**Figure 7:**
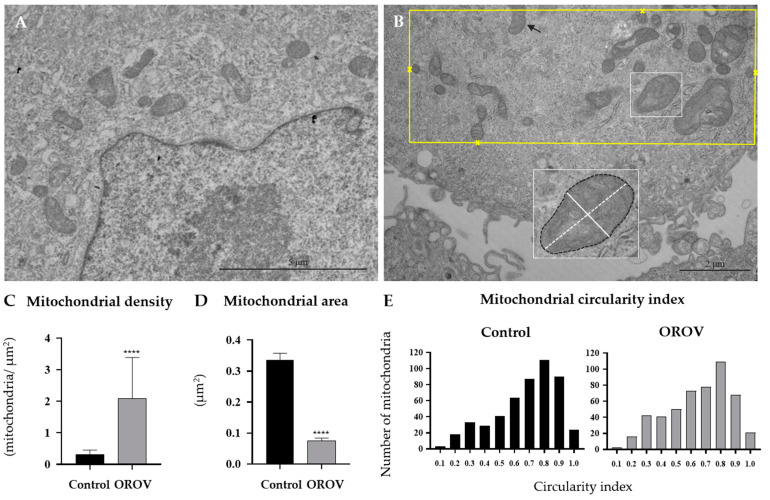
Morphometric analysis of the mitochondria. (**A**) Micrograph of the uninfected cell. (**B**) Micrograph of the OROV-infected cell culture to illustrate morphometric procedure. All mitochondria were counted, excluding those that were not fully within the field (black arrow). For the mitochondrial density of each micrography, the evaluated areas (yellow rectangle) were defined as the boundary surrounding the outermost mitochondria (yellow crosses). The cross-sectional area (black dashed line) and minor and major axes (continuous and dashed white line, respectively) were measured in all mitochondria (**C**) Mitochondrial density of 20 micrographs per group. Median: 0.29 mitochondria/μm^2^ (95% CI, 0.25–0.31)—control group), and 1.78 mitochondria/μm^2^ (95% CI, 1.27–2.3)—OROV-infected group. The data presented in panels (**C**,**D**) correspond to the micrographs totaling 500 mitochondria per group (control and OROV-infected). (**D**) Mitochondrial cross-sectional area. Median: 0.37 μm^2^ (95% CI, 0.32–0.36)—control group, and 0.08 μm^2^ (95% CI, 0.07–0.08)—OROV-infected group. **** *p* < 0.0001—Mann–Whitney test. (**E**) Distribution of the frequencies of mitochondrial circularity index. The y-axis indicates the number of mitochondria located within the circularity index range specified by the center bins on the x-axis. Median: 0.72—control group, and 0.68—OROV-infected group.

In comparison to the well-preserved mitochondria in uninfected cells (Figure 7A), OROV infection may be related with numerous ultrastructural alterations in these organelles, encompassing its localization, size, and morphology (Figure 7B). Given this context, initially, forty micrographs were analyzed to assess mitochondrial density. In the twenty images of the OROV-infected cell culture, a total of 558 mitochondria were quantified within a total area of approximately 355 μm^2^. In contrast, the control group exhibited 523 mitochondria in around 1980 μm^2^. These data reinforce the morphological findings of mitochondrial clustering during OROV infection. Consequently, in the evaluation by micrography, a higher mitochondrial density was observed, approximately 1.8 mitochondria/μm^2^, compared to 0.3 mitochondria/μm^2^ in the control group (Figure 7C).

To ensure a balanced comparison of mitochondrial area and circularity, 500 mitochondria were evaluated per group. Regarding mitochondrial area, the values recorded were 0.08 μm^2^ for the OROV-infected group compared to 0.37 μm^2^ in the control group (Figure 7D). In the infected cells, we also observed mitochondria with notably elevated cross-sectional areas, which were designated as giant mitochondria. However, the morphometric assessment revealed a reduction in the cross-sectional area of the mitochondria in these infected cells when compared to the control group (approximately fourfold). The mitochondria that appeared “giant” are, in fact, more comparable in size to the average values of mitochondria from the control group. Nonetheless, the morphological alterations observed in these larger mitochondria in infected cells, such as vacuolization and extremely elongate and irregular patterns (Figure 7B), indicate structural damage that was not present in uninfected cells (Figure 7A). A larger standard deviation was noted in the uninfected culture, highlighting the greater mitochondrial size pleomorphism in the control condition compared to the more homogeneous reduced sizes of mitochondria in the OROV-infected group.

The circularity index is assessed based on the apparent cross-sectional area of mitochondria and does not accurately reflect a direct sectional plane of their three-dimensional structure. Nevertheless, this cross-sectional area results from the rearrangement of these organelles in their cytoplasmic distribution due to physiological changes. Consequently, the comparative analysis between the control and infected cells becomes highly relevant. The circularity values observed for the groups were quite similar, with 0.68 for the infected group and 0.72 for the control. In the infected group, a slightly different behavior was observed, characterized by a modest increase in the number of mitochondria exhibiting lower circularity indices (Figure 7E).

### 3.4. OROV RNA Quantification from Vero Cell Culture Supernatants

To evaluate the susceptibility of Vero cells to produce OROV progeny, we quantified the number of copies of viral RNA in cell culture supernatants collected at 72 h p.i. The quantitative real time RT-PCR assay demonstrated a cycle threshold (Ct) equal to 18.52/18.28, suggesting the production of viral progeny.

## 4. Discussion

OROV infection has been linked to adverse pregnancy outcomes, including fetal loss, stillbirth, and neonatal microcephaly [13,27]. In addition, there has been a concerning connection between Oropouche fever and neurological manifestations, such as meningoencephalitis and Guillain–Barré Syndrome in convalescent patients, pointing to a potential long-term sequela of the infection [28]. Both scenarios may be associated with a novel reassortant lineage of OROV [10,16]. These findings underscore the urgent need to understand the OROV replication cycle and virus–cell interactions, for which in vitro systems play a crucial role.

Our results suggest that ATCC CCL-81 Vero cells are permissive to OROV infection, with significant CPEs throughout the infection period, including cell death. Despite their widespread use due to their susceptibility and permissiveness to various viruses [29], characteristics attributed to type I interferon production deficiency [30], Vero cells have not yet been established as the preferred model for OROV infection. In vitro studies have primarily been conducted using HeLa cells to evaluate virus entry [19], cellular mechanisms elicited by viral replication [31], and OROV egress [32].

One of the main effects observed in our morphological analysis was cytosolic and membrane fragmentation, resulting in multiple membrane-bound portions, which under in vivo circumstances would be engulfed by phagocytes or other adjacent cells [33]. In vitro, this phenomenon is more evident due to the abundance of small apoptotic bodies observed in the culture flask and corroborated by ultrastructural evaluation. This programmed cell death mechanism represents a typical cellular response to stress or injury but is also involved in viral infections [34]. It can be either stimulated, to facilitate viral egress and maintain late-stage pathogenesis while evading the immune system, or suppressed, to prevent cell death and enhance viral replication [35].

According to a study on HeLa cells, the OROV replication cycle has apoptosis as an important consequence of infection, requiring viral protein expression to trigger the mechanism [36]. As we observed OROV-like particles in MVBs within apoptotic bodies, it is possible to hypothesize that this represents primarily an infection mechanism of neighboring cells, as the viral particles may evade the immune system within membrane-bound cell fragments, which could then be internalized by other cells [37,38]. Furthermore, similar to apoptosis, both the autophagic and endosomal pathways exhibited significant alterations during infection. It was observed that, even though present in uninfected cells as well, electron-lucent vacuoles, such as MVBs containing membrane whorls and heterogeneous materials, may support packaging and trafficking steps in the morphogenesis of OROV, as previously demonstrated in SARS-CoV-2-infected Vero cells [39].

Changes in the morphology of the RER, such as the formation of whorls or myelin figures, are associated with cellular stress [40]. However, the inability to effectively counteract or at least mitigate viral infection provides a clear pathway for viral replication, resulting in the accumulation of proteins and potentially contributing to RER stress [41]. It is well established that the accumulation of misfolded proteins in the RER can activate the Unfolded Protein Response (UPR), and if this response does not resolve the protein overload, it can result in apoptosis, as previously observed [42]. Additionally, both RER thickening and the formation of cisternae are consistent with the response to viral replication, as shown for SARS-CoV-2 infection [43].

Mitochondria also displayed ultrastructural alterations and distribution influenced by the rearrangement of RER-derived membranes. Morphological changes in mitochondria are common to many processes involving metabolic stress and cell death, but are also associated with viral infections [44]. While mitochondria can naturally exhibit some pleomorphism and heterogeneity in size [45], visible under our TEM analysis, the diminished area observed during infection is often associated with fission, which is regulated by mitophagy, and with an increased metabolic demand or oxidative stress [46]. Likewise, the morphological shift of mitochondria to altered patterns, such as vesicular or swollen appearance, is a well-documented feature of stress response [47,48].

The close relationship between mitochondria and lipid bodies in the cell is associated with changes in the cellular lipid metabolism profile [49]. In our study, we observed a marked proliferation in lipid bodies in the OROV-infected cell culture compared to the control group. Indeed, the interaction between these organelles appears to be connected to lipophagy, a mechanism already highlighted as beneficial in the context of viral infections [50]. For positive-strand RNA viruses, particularly other arboviruses within the *Flaviviridae* family, alterations in cellular lipid composition have been well characterized, with these changes being associated with a more favorable cellular energy metabolism [51,52]. Additionally, lipids contribute structurally to the formation of new viral particles and are involved in the remodeling of replication complexes [53]. However, for negative-strand RNA viruses, while these lipid alterations have been described, their significance in viral replication processes remains to be fully elucidated [54].

In comparison to the aforementioned alterations, cytoskeletal rearrangements are less frequently discussed, despite their involvement in various viral mechanisms, from viral entry to egress [55]. Membrane protrusions, like filopodia, increase the contact area and form bridges between cells, as we observed, which may facilitate the cell-to-cell spreading of either viral particles or biomolecules [56,57]. In previous work by our group, filopodia emission with associated SARS-CoV-2 particles, throughout the mechanism of viral surfing, resulted in optimized infection, corroborating the ability of these extensions to modulate viral pathogenesis [58]. As we observed an intense cytoplasmic vesicle trafficking in OROV-infected cells, even in regions abundant with filopodia connecting cells, these projections can help cell-to-cell delivery, thus avoiding the extracellular environment.

In our analyses, with a mean diameter of 80 nm, the viral particles exhibited the morphological characteristics typical of OROV, and additional analyses will be conducted to specifically track the virus. Members of *Bunyavirales* generally bud from Golgi membranes, though the plasma membrane is an alternative site for assembly and budding [59]. We observed viral particles in Golgi cisternae, but their remarkable site was allowed by the intense reorganization of membranous structures. Specifically regarding orthobunyaviruses, one of the mechanisms proposed is that these viruses induce rearrangements in intracellular membranes, including the Golgi apparatus, and endosomal compartments, generating specialized vesicles that support viral particle maturation and assembly [60].

Structures like MVBs were described as sites of assembly and budding for OROV, and modifications in the endosomal sorting complex required for transport (ESCRT) occurred following the OROV infection of HeLa cells [61]. The detection of viral proteins within these formations supports the significance of alterations in the endosomal pathway [61], which are better comprehended for viruses such as Ebola (EBOV) and Marburg [62,63]. However, while the influence of these vacuolar structures [61] and clathrin-coated vesicles on the virus’ entry into cells [19] is better understood, little is known about the relationship between OROV infection and extracellular vesicles (EVs), which were widely observed in our study emerging from the cell surface, often in clustering.

A relationship between the severity and prognosis of diseases based on EVs has already been established [64]. For instance, Muratori and colleagues suggested a correlation between the secretion of microvesicle clusters, viral load, and HIV infection outcomes [65]. In our study, different formats of EVs were detected following OROV infection. In light of the immune evasion capabilities and trafficking of various biomolecules, including pro-inflammatory cytokines, as demonstrated in a study on EBOV [66], research on EVs is critical due to their potential therapeutic applications and roles as biomarkers [67].

The exact mechanisms underlying many stages of the OROV replication cycle remain poorly understood. Further complementary studies are essential to fully elucidate the roles of the crosstalk between apoptosis and autophagic and endosomal pathways, as well as membrane rearrangements, in the morphogenesis and dissemination of OROV. Nevertheless, our observations suggest that this in vitro model offers valuable insights into key aspects of the cellular response to OROV infection.

## Data Availability

Data are contained within the article.
